# Identification of Immunoreactive Peptides of Toxins to Simultaneously Assess the Neutralization Potency of Antivenoms against Neurotoxicity and Cytotoxicity of *Naja atra* Venom

**DOI:** 10.3390/toxins10010010

**Published:** 2017-12-25

**Authors:** Bing-Sin Liu, Wen-Guey Wu, Min-Han Lin, Chi-Han Li, Bo-Rong Jiang, Suh-Chin Wu, Chih-Hsiang Leng, Wang-Chou Sung

**Affiliations:** 1 National Institute of Infectious Diseases and Vaccinology, National Health Research Institutes, Miaoli 35053, Taiwan; bingsin@nhri.org.tw (B.-S.L.); hamlyn@nhri.org.tw (M.-H.L.); han@nhri.org.tw (C.-H.L.); jiangbj@nhri.org.tw (B.-R.J.); leoleng@nhri.org.tw (C.-H.L.); 2 Institute of Bioinformatics and Structural Biology, Department of Life Science, National Tsing Hua University, Hsinchu 30013, Taiwan; lswwg@life.nthu.edu.tw; 3 Institute of Biotechnology, Department of Medical Science, National Tsing Hua University, Hsinchu 30013, Taiwan; scwu@mx.nthu.edu.tw

**Keywords:** *Naja atra*, antivenom, neutralization, assessment, immunoreactive peptide, ELISA

## Abstract

Assessing the neutralization capability of nonlethal but medically relevant toxins in venom has been a challenging task. Nowadays, neutralization efficacy is evaluated based simply on the survival rates of animals injected with antivenom together with a predefined dose of venom, which can determine potency against neurotoxicity but not validate the capability to neutralize cytotoxin-induced complications. In this study, a high correlation with in-vivo and in-vitro neutralization assays was established using the immunoreactive peptides identified from short-chain neurotoxin and cytotoxin A3. These peptides contain conserved residues associated with toxin activities and a competition assay indicated that these peptides could specifically block the antibody binding to toxin and affect the neutralization potency of antivenom. Moreover, the titers of peptide-specific antibody in antivenoms or mouse antisera were determined by enzyme-linked immunosorbent assay (ELISA) simultaneously, and the results indicated that Taiwanese bivalent antivenom (BAV) and Vietnamese snake antivenom-Naja (SAV-Naja) exhibited superior neutralization potency against the lethal effect of short-chain neurotoxin (sNTX) and cytotoxicity of cardiotoxin/cytotoxin (CTX), respectively. Thus, the reported peptide ELISA shows not only its potential for antivenom prequalification use, but also its capability of justifying the cross-neutralization potency of antivenoms against *Naja atra* venom toxicity.

## 1. Introduction

*Naja atra*, also known as Formosa or the Chinese cobra, is a highly venomous snake widely distributed in Pan-Asia and accounts for approximately 11% and 17%, respectively, of snake envenomings in Taiwan and China annually [1,2]. *N. atra* venom contains abundant three-fingered toxins (3FTXs) that contribute significantly to neurotoxic manifestations and severe local tissue damage in snakebite victims [3]. Clinically, intravenous administration of antivenom has been recommended for the treatment of cobra-bitten victims. Based on regulatory guidelines, the neutralization potency of antivenom, in terms of median effective dose (ED_50_) [4] or Tanaka unit (TU) [5], was determined by the amount of antivenom that could neutralize the designed dose of snake venom and rescue half or total animals tested, particularly mouse species. Although such an *in-vivo* animal assay could directly reflect the neutralization capability of antivenom against the lethal toxins of crude venom, it comes with the restriction of having to estimate the protective efficacy of sublethal but toxic venom components that lead to tissue necrosis or necrotizing fasciitis in cobra-bitten victims [2,3]. In this regard, developing an effective in-vitro approach that could thoroughly evaluate the neutralization potency would not only be of great benefit for better qualification control of antivenom, but also minimize the use of animals based on ethical considerations.

Since the therapeutic effectiveness of antivenom relies on the content of toxin-specific protective antibodies within, an immunological approach, such as enzyme-linked immunosorbent assay (ELISA) [6,7] or antivenomics [8,9,10], could be a way to evaluate the neutralizing potency against specific toxin activity. The process of developing antivenomics, with the intent of profiling the binding capacity of antivenom to venom components, involves affinity chromatography and proteomic characterization. By quantifying the immune-captured principal toxins, the potency of antivenom can be assessed because of its high correlation with *in-vivo* neutralization assay [10,11,12]. However, the laborious procedure and the expensive instruments have restricted its wider application [13]. 

In contrast, ELISA is a simple and sensitive approach that can directly quantify toxin-specific antibodies by the colorimetric method, and the results have been correlated with in-vivo neutralization efficacy [7,14]. To this end, abundant purified toxin is required, and the preparation of coating antigen can be challenging and time-consuming. In light of the antibody–antigen interaction being restricted to the unique residues involved in contact, these antigenic determinants, also known as B-cell epitopes, could probably be substituted for venom toxins as coating antigens for ELISA-based potency evaluation. Recent discoveries using peptide array technology have identified a number of B-cell epitopes of venom toxins [15,16,17,18,19,20]. In addition, a series of immunoprofiling studies further indicated that antivenom antibodies attempt to bind to functional domains of toxins, such as basic phospholipase A_2_ (PLA_2_) from *Bothrops jararacussu* venom and type 1 α-neurotoxins from venom of the Elapid snake family, thereafter neutralizing the toxicity by sterically blocking their interaction with the cellular counterparts [21,22]. In line with this concept, the B-cell epitope-containing residues associated with toxin activity can be a potential ELISA coating antigen to quantify potent antibodies for antivenom potency evaluation. 

Short-chain neurotoxin (sNTX) and cardiotoxin/cytotoxin (CTX) are two medically important classes of toxins in venom of *N. atra*, and snakes inhabiting different geographic regions may contain multiple isoforms of each classes in their venom composition [23]. Both sNTX and CTX share similar three-finger-type structures that contain four disulfide bonds and form three beta-sheet loops projecting from the central core. However, in contrast to the neurotoxic activity of sNTX, CTX induces severe cytotoxic syndromes in cobra-bitten victims [24]. In the study, two immunoreactive peptides containing critical epitopes associated with either neurotoxic or cytotoxic activity were identified by overlapped peptide array coupled with the antivenom antibodies. The titers of peptide-specific antibody quantified by ELISA showed a high correlation with either in-vivo or in-vitro neutralization assay results. The usefulness of peptide ELISA was also verified in an extensive study using mice antisera and a similar correlation was noted. Our results demonstrate that the developed peptide-based ELISA is an effective preclinical assay that is able to evaluate the neutralization potency of antivenom against either lethal or cytotoxic activity induced by *N. atra* venom.

## 2. Results and Discussion

### 2.1. Determination of Major Toxic Component of N. atra Venom 

To confirm medically relevant targets in the tested *N. atra* venom, a toxicovenomic approach [25,26] based on antivenomics and in-vivo toxicological analysis was employed in the study. Initially, the immunocapturing profile of antivenom against venom components was determined using the antivenomic approach (Figure S1). Based on the results of affinity chromatography and protein identification (Table S1), there was a remarkable difference in the quantity of sNTX, CTXs and acidic PLA_2_ molecules captured by bivalent antivenom (BAV), snake antivenom-Naja (SAV-Naja) and neuro polyvalent snake antivenom (NPAV), respectively, as shown in Table 1. By contrast, the other venom proteins were well-trapped on affinity columns. Furthermore, in-vivo animal assays were conducted to measure the lethality (median lethal dose, LD_50_) of these incompletely retained fractions, and results showed that sNTX (P60770, UniProt, LD_50_ = 0.23 μg/g,) was more lethal than CTXs (LD_50_ = 2.12 μg/g) and even crude venom (LD_50_ = 0.67 μg/g), while PLA_2_ was devoid of lethal effect on mice even under a challenged dosage of 50 μg protein per mouse (19–21 g). Apart from the lethal dosage determination, mice injected intraperitoneally with 5 × LD_50_ sNTX or crude venom developed similar symptoms of flaccid paralysis, which resulted in sudden death within an hour. Overall observations shown here suggest that sNTX is a major lethal component in *N. atra* venom, which is in agreement with other investigations showing that α-neurotoxin was the main toxin to cause death in mice [27,28].

**Figure 1:**
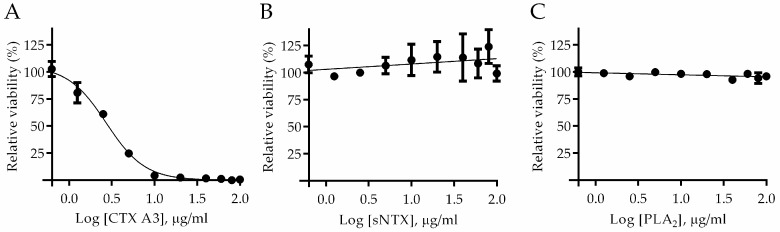

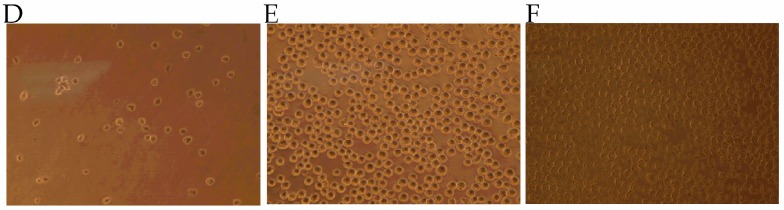
Cytotoxin exhibits intense cytotoxic effect as compared to other major venom toxins. Here, 1 × 10^5^ HL-60 cells were incubated with toxins of (**A**) CTX A3, (**B**) sNTX, and (**C**) PLA_2_ in various concentrations, and the cytotoxicity of toxins was determined. The assay was performed in triplicate and the viability of toxin-treated cells is represented as mean ± SD. The images represent the morphology of cells incubated with 40 μg/mL of (**D**) CTX A3, (**E**) sNTX, and (**F**) PLA_2_.

In contrast, there were no evident symptoms in mice administered isolated CTXs (5 × LD_50_), and the live-duration was obviously longer, over an hour or more than mice injected with sNTX molecule. In general, CTX is a non-enzymatic three-fingered toxin that contains abundant basic residues but shows amphiphilic properties. In addition, CTX was found to interact with negatively charged glycosaminoglycans on the membrane, and caused cell necrosis by disrupting the integrity of cell membranes via pore formation [29,30]. Considering the unique cell penetration activity [31], in-vitro cell-based penetration assay was implemented to characterize the toxic effect of the CTX molecule. Figure 1A shows the sigmoid growth curve of toxin-treated cells, and results indicate that treatment of CTX analogs would induce cell mortality in a dose-dependent manner. Based on cell viability, the half-maximal inhibitory concentration (IC_50_) of CTX A3 was determined to be 2.72 μg/mL, which is significantly lower than the value of CTX mixture (6.82 μg/mL) and other CTX analogs (Figure S2). In contrast to CTX molecules, no apparent loss of viability was observed on sNTX- and PLA_2_-treated cells [32], shown by the data in Figure 1B,C. From the findings of animal and cell-based toxicity assays, sNTX and CTX A3 are the critical elements responsible for venom lethality and cytotoxicity.

### 2.2. Identification of Immunoreactive Peptides of Toxins

**Figure 2:**
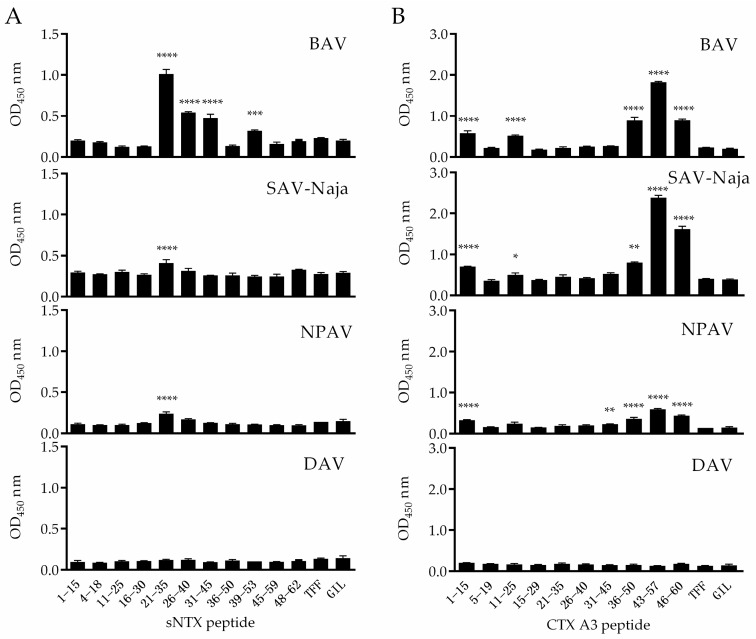

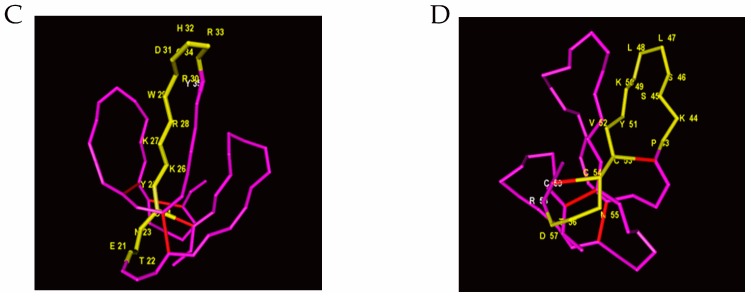
The recognition of antivenom toward synthetic peptides derived from sNTX and CTX A3 toxins is shown. The overlapping peptides of (**A**) sNTX and (**B**) CTX A3 were coated on the ELISA plate, and the reactivity of peptide-specific antibodies in antivenom of BAV, SAV-Naja, NPAV and DAV was measured. TFF and GIL peptides were used as negative controls in the assay. Significant difference calculated with the two-tailed Student’s *t*-test is marked by asterisks (peptide vs. TFF (control), **** *p* < 0.0001, *** *p* < 0.001, ** *p* < 0.01, * *p* < 0.05). The immunoreactive peptides of sNTX_21−35_ and CTX A3_43–57_ are marked in yellow in the crystal structure of (**C**) sNTX (PDB: 1COD) and (**D**) CTX A3 (PDB: 2BHI), respectively. The part marked in red represents the disulfide bridges of toxin. Images were generated using Cn3D software [37].

In the study, a total of 21 peptides (Table S2) corresponding to mature sNTX and CTX A3 sequences were synthesized and individually coated on wells to screen out potential immunoreactive candidates recognized by antivenoms. Considering the contact area of antibody on antigen is about 600–1200 Å^2^ [33], each peptide was designed with 15 continuous residues and 10 overlapping amino acids. Three commercial antivenoms, BAV, NPAV and SAV-Naja, were used to probe epitopes from the coating peptides, and an antivenom against *Deinagkistrodon. acutus* (DAV) was applied as the control in this screening assay. Figure 2A shows the reactivity of antivenoms against synthetic peptides derived from the sNTX sequence, and the results indicate that antivenoms consistently reacted with the peptides of sNTX_21–35_, the sequence of which corresponded significantly with the loop II region of type 1 α-neurotoxins (Figure 2C), while the adjacent peptide sNTX_16–30_ was barely recognized. Therefore, it was hypothesized that the sequence of DHRGY (residues 31–35 of sNTX protein) was the B-cell epitope within sNTX_21–35_. It has been noted that DHRGY is highly conserved in type 1 α-neurotoxins [20,28] and was found to be the binding motif with nicotinic acetylcholine receptors [34,35,36], which emphasizes that this consensus sequence might be endowed with the lethal activity of sNTX. 

To identify the immunoreactive peptide of CTX A3, the selected cobra antivenoms were found to react with peptides of CTX A3_36–50_, CTX A3_43–57_ and CTX A3_46–60_, which majorly correspond with the loop III region of the CTX molecule, as shown in Figure 2B,D. Among the peptides tested, the reactivity of antibody with CTX A3_43–57_ was more dominant than the adjacent peptides, which suggests that the sequence of SSLLVKY (residues 45–51 of CTX A3) was the potential B-cell epitope. Similar to other cytotoxins, CTX A3 contains hydrophobic LL residues flanked by polar amino acids and forms a cytolytic region on the tip of loop III, which enables the toxin to anchor the cell membrane and perturbs its integrity [36,38,39,40]. The multiple sequence alignment also revealed that a high amount of conserved residue was clustered in the C-terminal region of these cytotoxic 3FTXs. As the peptide contained residues related to toxic activity, CTX A3_43–57_ was selected as the ELISA coating antigen to quantify the biocorrelation of peptide-specific antibody titer with antivenom potency. Taken together, these results indicate that there is a linear B-cell epitope located in the peptides of sNTX_21–35_ and CTX A3_43–57_. These immunoreactive peptides were shown to be cross-recognized by heterologous antivenoms raised by different cobra venoms, probably due to the high similarity of certain residues. In addition, the structural features indicate that both sNTX_21–35_ and CTX A3_43–57_ are crucial peptides on the corresponding toxins; the usefulness of these immunoreactive peptides for neutralization potency assessment was evaluated in the following study.

### 2.3. Specificity of Peptide-Specific Antibodies

**Figure 3:**
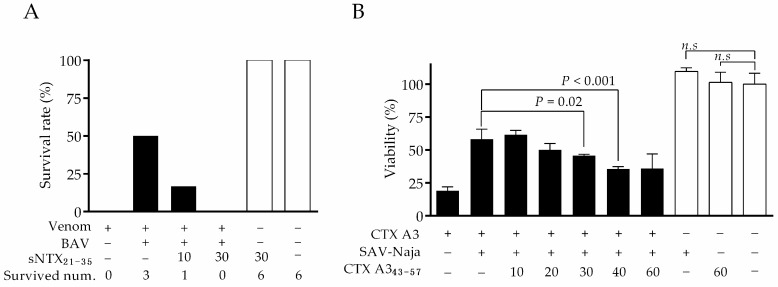
Analysis of the peptide-blocking effect on antivenom neutralization potency. Changes in the percentage of (**A**) animal survival rate (*n* = 6 per group) and (**B**) cell viability (1 × 10^5^ cells per assay) revealed that synthetic peptides of sNTX_21–35_ and CTX A3_43–57_ blocked the binding of antibody with the toxicity sites of toxins, which inhibited antivenom potency against venom toxicities. Significant differences were observed between groups comparing cellular viability by one-way analysis of variance followed Fisher’s least-significant difference test.

The principle of neutralization is based on the binding of antivenom antibodies to the toxicity sites of toxins. To clarify whether peptide-specific antibodies can neutralize a toxin, the excess amount of peptide was added to antivenom to compete with corresponding toxin molecules for binding with antibodies, and the changes in neutralization potency were monitored, as described in the experimental section. In the study, BAV and SAV-Naja were selected to justify the competition effect, since they contain more antibodies reactive to the sNTX_21–35_ and CTX A3_43–57_ peptides, respectively. As the results of the in-vivo neutralization assay shown in Figure 3A demonstrate, the addition of sNTX_21–35_ peptide obviously reduced BAV neutralization efficacy against venom lethality, as indicated by a decrease in the survival rate of mice after venom challenge. When a 30-fold molar ratio of sNTX_21–35_ peptide was spiked, the neutralization efficacy of BAV was completely inhibited and no mice survived after venom challenge, which provides evidence that sNTX_21–35_ contains a neutralizing epitope similar to that of sNTX toxin. It was noted that no lethal effect in mice injected with a 30-fold molar ratio of sNTX_21–35_ peptide alone was observed. In contrast, the irrelevant peptide of sNTX_1–15_ did not inhibit neutralization potency (data not shown). 

The blocking effect of CTX A3_43–57_ peptide on SAV-Naja was analyzed by the in-vitro cell assay. After blocking with various concentrations of CTX A3_43–57_ peptide, the neutralization efficacy of SAV-Naja dropped in a dose-dependent manner, and the neutralization potency of SAV-Naja was inhibited by nearly 30% when the highest amount of CTX peptide was spiked, as shown in Figure 3B. In parallel, the experiment control of either CTX A3_43–57_ peptide or antivenom alone showed no evident cytotoxicity to the cell. Overall, the competition assay confirmed that the epitopes recognized by peptide-specific antibodies were critical toxic sites, which also highlighted that these peptide-specific antibodies were likely to be the neutralizing antibodies in antivenom.

### 2.4. Correlation between Neutralization Potency and sNTX_21–35_ Antibody Titer

**Figure 4:**
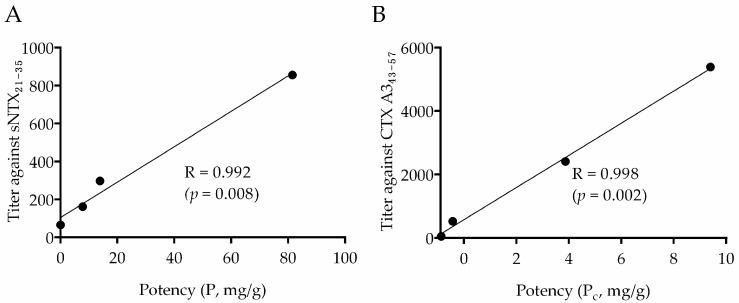
Correlation plots of the neutralization potencies and ELISA antibody titers of commercial antivenoms against (**A**) sNTX_21–35_ and (**B**) CTX A3_43–57_ peptides.

In light of sNTX_21–35_ and CTX A3_43–57_ exhibiting potential neutralization epitopes, it was of particular interest to investigate the biocorrelation between the amounts of peptide-specific antibodies and the neutralization potency of antivenom. On top of the investigation, in-vivo neutralization assay was performed to determine the potency of antivenoms against venom lethality. As the data in Table 2 show, BAV exhibited higher neutralizing efficacy (potency (P) = 81.5 mg venom/g antivenom) than SAV-Naja (P = 13.9 mg/g) or NPAV (P = 7.8 mg/g). It is noted that the neutralization potency of NPAV against *N. atra* venom was quite similar to the other investigation [41]. Subsequently, sNTX_21–35_-specific antibody titers, calculated from peptide ELISA using serial diluted antivenoms, were compared with corresponding *in-vivo* neutralization potency (P), and a high correlation was seen, with a correlation coefficient (R) value of 0.992, as shown in Figure 4A. In contrast, no significant correlation was found with other peptide-specific antibody titers. Compared to BAV, there was a smaller amount of sNTX_21–35_-specific antibody in antivenoms of SAV-Naja and NPAV. This was expected, since only a small amount of sNTX (e.g., 4.2–9.2% type 1 neurotoxin) was included in *N. kaouthia* venom [42]. Such high coincidence emphasizes the feasibility of directly quantifying the sNTX_21–35_-specific antibody titer as a way to justify the neutralization potency of cobra antivenoms against the lethality of *N. atra* venom.

### 2.5. Correlation between Cytotoxic Inhibition Potency and CTX A3_43–57_ Antibody Titer

**Figure 5:**
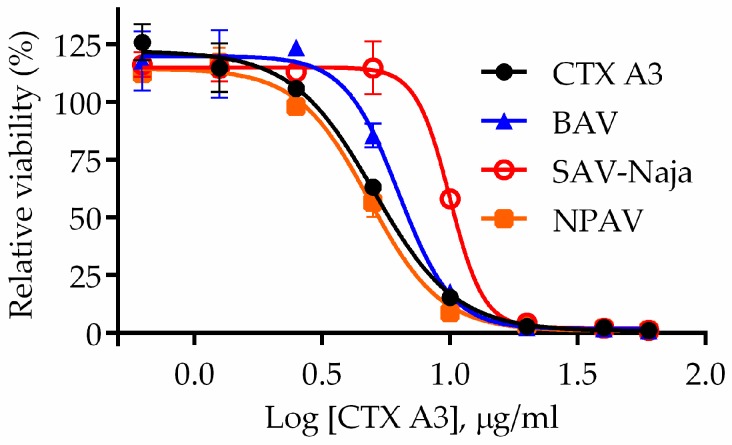
In-vitro cell-based neutralization assay. The sigmoid curve of cell viability was obtained by treating cells with a mixture of varying doses of CTX A3 protein together with either a constant amount of BAV (▲), SAV-Naja (○) or NPAV (■). The cells incubated with toxin alone were utilized as controls (●) in the assay. The neutralizing potency of antivenom against cytotoxicity was calculated by the equation described in the experimental section. Results were expressed as mean ± SD from three independent experiments done in triplicate.

In parallel, an in-vitro cell-based assay was employed to evaluate the neutralization potency of antivenom against CTX A3, the most cytotoxic component of *N. atra* venom, and the results are shown in Figure 5. Surprisingly, SAV-Naja (P_c_ = 9.40 mg/g) was approximately three times more potent in neutralizing CTX A3 than BAV (P_c_ = 3.87 mg/g). However, the neutralization potency of NPAV against CTX A3 was nearly negligible compared to that of DAV (negative control). Given genomic and venomic reports, venoms of *N. atra* and *N. kaouthia* contain similar classes of CTX molecules [43]. SAV-Naja, however, was much more potent than BAV in neutralizing the toxin cytotoxicity, which was probably due to the higher amount of CTX homologs in *N. kaouthia* venom from Vietnam, which was used as the immunogen for SAV-Naja production. By plotting the potency values with the ELISA CTX A3_43–57_-specific antibody titers, a high correlation was established, with a coefficient value of 0.998, as shown in Figure 4B. Currently, the method to evaluate the potency of antivenom against venom cytotoxicity has not been standardized. Considering the convenience of assay preparation and the high correlation with the bioassay, peptide ELISA could be an applicable approach to evaluate the potency of antivenom against venom cytotoxicity.

### 2.6. Assessment of the Neutralizing Capability of Mouse Antiserum by Peptide ELISA

**Figure 6:**
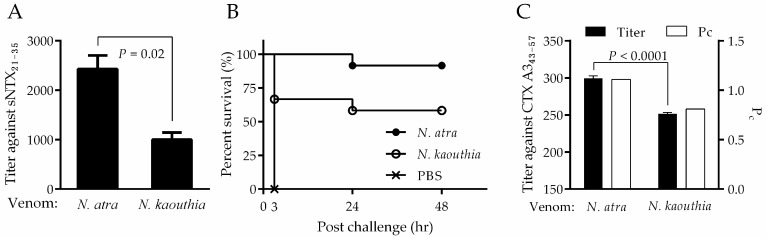
Evaluation of the immunogenicity of two species-related cobra venoms in mice. (**A**) Detection of antibody titers against peptide of sNTX_21–35_ in mice immunized with either *N. atra* or *N. kaouthia* venom. (**B**) The survival rate of venom-immunized mice after challenging with *N. atra* venom (3 × LD_50_). (**C**) Correlation of CTX A3_43–57_-specific antibody titers with neutralization potency (P_c_) of pooled antiserum obtained from in-vitro cell assay. The antibody titer is expressed as mean ± SD.

Prior to proceeding with horse immunization for the production of antivenom, it was useful to perform a pilot study aimed at optimizing the efficacy and safety of the immunogen. Considering the ease of operation and its consistency in immune response, the mouse seemed to be a suitable model for pretesting. However, the amount of mouse antiserum was limited for subsequent neutralization assays. In the study, two groups of BALB/c mice (*n* = 12) were immunized with either *N. atra* or *N. kaouthia* venom, and the antiserum was used to investigate the correlation of peptide-specific antibody titers with neutralization potency. Through the peptide ELISA, pooled antiserum from mice immunized with either *N. atra* venom or *N. kaouthia* venom was determined to have sNTX_21–35_-specific antibody titers of 2430.67 ± 271.49 and 1000.28 ± 140.89, respectively (Figure 6A). Such discrepancy in peptide-specific antibody titer was probably due to more abundant sNTX in *N. atra* venom (Figure S3), which in turn led to better neutralization potency against venom lethality, as the survival rate of *N. atra* venom-immunized mice (91%; 11/12 mice survived) was higher than that of *N. kaouthia* venom-immunized mice (58%; 7/12) (Figure 6B). Additionally, a significant difference in CTX A3_43–57_-specific antibody titer was found between the pooled serum from mice immunized with *N. atra* venom (299.30 ± 3.30) and *N. kaouthia* venom (251.67 ± 1.70). Subsequently, the pooled mouse serum was evaluated for neutralization potency against cytotoxicity induced by CTX A3. As the data in Figure 6C show, the serum raised by *N. atra* venom (P_c_ = 1.2 mg/g) was approximately twice as potent in neutralizing CTX A3 as that raised by *N. kaouthia* venom (P_c_ = 0.67 mg/g). These results suggest that: (1) either *N. atra* or *N. kaouthia* venom would elicit a similar antibody response against sNTX_21–35_ and CTX A3_43–57_ peptides in mice, and (2) the neutralizing capability of pooled antisera against cytotoxicity was in proportion to the stimulated CTX A3_43–57_ antibody titers. These findings were consistent with the results of the present study, where we have demonstrated a good correlation between these tests when horse antivenoms were analyzed.

## 3. Conclusions

Toxicovenomic analysis of venom of *N. atra* reveals that a major presence of 3FTXs molecules is responsible for the predominant toxic activities of cobra venom. With the aid of peptide mapping, the CTX A3 epitope (CTX A3_43–57_) and sNTX epitope (sNTX_21–35_) were identified as potential coating antigens for the assessment of potency against cytotoxic and lethal toxic effects, respectively. The results of multiple sequence alignments demonstrate that these two toxin epitopes contain conserved residues, which have been reported to be associated with toxic activities of 3FTX. In the study, a quantitative ELISA assay using toxin epitopes as coating antigens was established to quantify the peptide-specific antibody titer in antivenoms. By validating with three anti-cobra antivenoms and mouse antisera, a high correlation was found between peptide-specific antibody titer and corresponding in-vivo and in-vitro assay results. Among the antivenoms tested, the data further show that BAV and SAV-Naja exhibited the highest potency in neutralizing lethal and cytotoxic activity. To the best of our knowledge, this is the first study to present the superiority of SAV-Naja for cross-neutralizing CTX of *N. atra*, despite its being raised by *N. kaouthia* venom.

Snake envenoming remains a severe public health problem in tropical and subtropical countries. Antivenom administration is the recommended treatment, with proven therapeutic effectiveness. Due to a lack of commercial interest, manufacturing region-specific antivenom could be challenging, and the shortage of antivenoms has become a global crisis owing to their discontinuation. In addition to developing a novel antivenom with a broad neutralization spectrum, selecting the appropriate antidote with para-specificity (i.e., the capability to cross-neutralize venom toxicity) from the existing antivenoms generated from venoms of closely related species might be another way to solve this issue. In the study, we showed that peptide ELISA is a promising in-vitro assay that not only quantifies the amount of neutralization antibody in antivenoms, but also is capable of determining the cross-neutralization potency of heterologous antivenoms against *N. atra* venom. Recently, the immunogenic properties of sNTX_21–35_ and CTX A3_43–57_ were tested in a follow-up study, and the neutralization potency of antisera is currently under investigation, which could benefit the designation of a broad-spectrum antivenom.

## 4. Materials and Methods

### 4.1. Chemicals and Materials

All chemicals used in this study were purchased from Sigma-Aldrich (St. Louis, MO, USA). The medium and antibodies used for cell culture were purchased from GE Healthcare Life Sciences. The cell viability assay was performed using Cell Counting Kit-8 (CCK-8; Dojindo Molecular Technologies, Rockville, MD, USA). *N. kaouthia* venom, a pool of snake venoms from Thailand, was obtained from Latoxan (France). *N. atra* venom was a pool of venoms collected from a snake farm in Taiwan and subsequently vacuum dried, filtered, and stored at −20 °C. Commercial bivalent antivenom (batch 61-06-0002, expiry date 23/12/2019), obtained from the Taiwan Centers for Disease Control (TWCDC), and was prepared from the plasma of horses hyperimmunized with venoms of *Bungarus multicinctus* and *N. atra*. Viper antivenom obtained from TWCDC was produced from the plasma of horses hyperimmunized with *Deinagkistrodon acutus* venom (DAV; batch 62-06-0006, expiry date 23/12/2019). SAV-Naja (batch 34, expiry date 14/08/2016), an antivenom product, was manufactured by the Institute of Vaccines and Medical Biologicals (Nha Trang, Vietnam) using venom of *N. kaouthia*, as shown on the datasheet. Neuro polyvalent antivenom (lot NP00115, expiry date 24/03/2020), a kind gift from Dr. Wu (National Tsing Hua University, Hsinchu City, Taiwan), was produced at the Queen Saobhava Memorial Institute (Bangkok, Thailand) from the plasma of horses immunized with venoms of *N. kaouthia*, *Ophiophagus hannah, B. candidus, and B. fasciatus*.

### 4.2. Preparation of Aantibody-Immobilized Columns

Twenty milligrams of antivenom antibodies dissolved in 1 mL of coupling buffer was slowly loaded into a pre-equilibrated HiTrap™ NHS-activated HP column (1 mL; GE, Uppsala, Sweden) and incubated for 1 h at room temperature. The column was then blocked with ethanolamine (0.5 M in 0.5 M NaCl, pH 8.3) and immediately washed with sodium acetate buffer (0.1 M in 0.5 M NaCl, pH 4.0) to remove the unbound material. The blocking and washing steps were repeated three times for the column preparation. A bicinchoninic kit (BCA; Thermo Scientific™ Pierce™) was used to quantify the amount of immobilized antibody by comparing the amount of protein in flow-through fraction with the raw material.

### 4.3. Immunoaffinity Analysis of Venom Proteins

The affinity analysis followed the procedure developed by Fahmi et al. [44] with slight modifications. To avoid sample degradation, the analytical procedures were performed at 4 °C with fast protein liquid chromatography (GE Healthcare, Piscataway, NJ, USA) equipped with fraction collector (GE Healthcare) and optical detector (UV 280 nm). Five hundred micrograms of venom dissolved in 1 mL of phosphate buffer was injected into the antibody-immobilized affinity column at a flow rate of 0.3 mL/min, followed by a washing step to remove the unbound protein using 10 mL of phosphate-buffered saline (PBS) (pH 7.5). Subsequently, the retained components were eluted with acetic acid (10%, v/v) at a flow rate of 1 mL/min for another 10 min. The flow-through and elution fractions were collected for further antivenomic analysis. The extent of protein binding to the antivenom-immobilized column was calculated based on the peak areas of retained (R) and non-retained (NR) fractions in the RP–HPLC chromatography (UV 280 nm), and the percentage of retained protein (%) was quantified using the equation as follows: % retained protein = 100 − [100 × (NR/(R + NR))].

### 4.4. Reverse-Phase HPLC Separation of Venom Proteins

Fractionation of venom components was performed in a high-performance liquid chromatography (HPLC) system (Alliance 2695; Waters, Milford, MA, USA) equipped with a dual absorbance ultraviolet detector (Model 2487). The venom analyst dissolved in distilled water was subjected to reverse-phase column chromatography (250 × 4.6 mm, 5 μm particles with 300 Å pore size; Jupiter C18, Phenomenex, Torrance, CA, USA) and eluted at 0.8 mL/min flow rate with two mobile phases (mobile phase B: 0.1% trifluoroacetic acid (TFA); mobile phase C: 100% acetonitrile (ACN/0.1% TFA) following the gradient: 2% C for 5 min, 2–10% C for 2 min, 10–16% B for 6 min, 16–28% B for 2 min, 28–65% B for 37 min, 65–80% B for 3 min, and 2% C for 10 min. The absorbance of the eluate was monitored at 215 and 280 nm, and the peak-containing fractions were pooled, lyophilized, and stored at −20 °C for further analysis.

### 4.5. Trypsin Digestion and Protein Identification

Protein identification was performed following the procedure described in a previous study [23]. Thirty micrograms of lyophilized sample was resuspended with 100 mM ammonium bicarbonate buffer (AB; pH 7.8), followed by denaturation with dithiothreitol (20 mM in AB buffer) and iodoacetamide (50 mM in AB buffer) in sequence. The denatured sample was mixed with trypsin (Promega) with a weight ratio of 1:20 and proceeded to sample digestion at 37 °C overnight. A Synapt HDMS ESI–Q–TOF mass spectrometer connected to a nanoACQUITY UPLC system (Waters) was used to identify the protein in venom digest. Five microliters of sample digest was injected and then separated with a C18 column (75 μm id × 10 mm, 1.7 μm bead size; Waters). Mobile phase A consisted of 0.1% formic acid (FA; Sigma) in deionized water, and mobile phase B consisted of 0.1% FA in 100% ACN. The flow rate was set at 200 nL/min with a linear gradient from 1% B to 50% B for 30 min, then increased to 65% B for 10 min, maintained at 65% B for 5 min, and back to 5% B for column condition. The survey scan was from 400 to 1600 *m*/*z* and the MS/MS scan was from 50 to 1990 *m*/*z*. The threshold to switch from MS to MS/MS was 40 counts, and the switch back was until the signal was under 10 counts or after 2.4 s. 

For protein identification, Masslynx 4.0 Global ProteinLynx was applied to transfer the raw data of MS/MS spectra to peak list (PKL). UniProt on MASCOT server (version 2.4.1; Matrix Science, London, UK) was used for the database search. The mass tolerance was set within 0.2 Da for both precursor and product ions. Carbamidomethyl and oxidization were used for fixed and variable modification, respectively. One trypsin miscleavage was allowed in the MS/MS ion search. Only proteins with scores >30 and *p* value <0.05 were considered significant hits. At least one unique peptide was considered as the minimum requirement for confident protein identification.

### 4.6. Animal Experiments

#### 4.6.1. Animals

ICR (CD-1, body weight 19–21 g) and BALB/c (6–8 weeks old) mice, purchased from BioLASCO Taiwan Co., Ltd., Nangang, Taiwan, were housed in the National Health Research Institutes (NHRI) animal center in accordance with a protocol (NHRI-IACUC-105108A) approved by the Institutional Animal Care and Use Committee of NHRI. Mice were allowed ad libitum access to food and water.

#### 4.6.2. Determination of the Lethality of *N. atra* Venom and Purified Toxins

The procedure to determine the lethality of toxin analyst was conducted following the protocol developed by Villalta et al. [45] with minor modifications. Groups of ICR mice (*n* = 6) were injected intraperitoneally (ip) with 200 μL of serial-diluted toxin solution. The survival rate of mice was recorded 48 h after the venom injection, and the toxicity was expressed as the LD_50_ (μg/g, toxin amount/mouse weight) corresponding to the quantity that caused half the mouse deaths in the assay, which was calculated by the trimmed Spearman–Karber method [46].

#### 4.6.3. In-Vivo Neutralization Assay

To evaluate the neutralization potency against *N. atra* venom, groups of six ICR mice were challenged with a mixture containing 5 × LD_50_ of *N. atra* venom with various dilutions of antivenom. The mixture was preincubated at 37 °C for 30 min and centrifuged at 4000 *g* for 10 min to remove precipitated impurities. Then, 200 μL supernatant was injected ip into the ICR mice, and the survival rate was recorded for 48 h. The median effective dose (ED_50_) of antivenom was calculated by the trimmed Spearman–Karber method and defined as the weight ratio of venom (mg) to antivenom (g) at which half the mice survived under the venom challenge. The effectiveness of antivenom was also expressed in terms of neutralization potency (P), calculated according to Morais et al. [47]. Owing to various protein concentrations of antivenom products, the potency was further normalized and expressed as milligram of venom neutralized per gram of antivenom protein.

#### 4.6.4. Venom Immunization

Immunization followed the protocol described in a previous study [23]. In brief, the lyophilized venom was reconstituted in sterilized PBS and then detoxified with glutaraldehyde (0.25% v/v) at 4 °C overnight. Groups of 12 mice were primed intramuscularly (im) with 50 μg of detoxified venom formulated in complete Freund’s adjuvant. Continuous boosting was done two times with the same amount of antigens, formulated in incomplete Freund’s adjuvant at 2-week intervals. Blood was collected by tail vein bleeding 2 weeks after the final immunization. The acquired serum was decomplemented (30 min at 56 °C) and then stored at −20 °C until use.

### 4.7. Cytotoxicity of Toxins

Human promyeloblast cells (HL-60) were from American Type Culture Collection. A total of 1 × 10^5^ HL-60 cells suspended in 100 µL of RPMI-1640 complete medium (Hyclone, GE) supplemented with 10% heat-inactivated fetal bovine serum and 1% penicillin/streptomycin antibiotics were seeded in 96-well microplates (Corning, Corning, NY, USA). Cells were cultured in a humidified atmosphere at 37 °C with 5% CO_2_ and then treated with various concentrations of snake toxins (0.625–100 μg/mL). After 4 h, 10 μL of CCK-8 solution was added to each well and the plate was further incubated for 4 h for measurement of cell viability by determining dehydrogenase activity in cells. Absorbance at 450 nm was measured by microplate reader (Sunrise; Tecan, Mannedorf, Switzerland) and the viabilities of untreated cells and medium alone were 100% and 0%, respectively. All treatments were performed in triplicate and the results were fitted to a dose-dependent sigmoid curve with variable slope using GraphPad Prism software (GraphPad, La Jolla, CA, USA). Cytotoxicity was expressed as the IC_50_ value (μg/mL) corresponding to the concentration of toxin that caused 50% cell death.

### 4.8. In-Vitro Neutralization Assay against Cytotoxicity

To investigate the antivenom neutralizing capability against the cytotoxicity, various amounts (20, 16, 12, 8, 4, 2, 1, 0.5, 0.25, 0.125 and 0.0625 μg/mL) of purified CTX A3 were pre-incubated with 100 μg of antivenoms in 100 μL of complete medium at 37 °C for 30 min. The antivenom–toxin mixture was then applied to 100 μL of 1 × 10^5^ of HL-60 cells in the microwells and the viability of cells was determined by the assay described above after 8 h of co-culturing. The efficacy (P_c_) of antivenoms was estimated by the following equation:

P_c_ = V × [IC_50_(a_1_) − IC_50_(a_0_)]/(a_1_ − a_0_), (1)

where V is the volume of the well, a is the amount (μg) of antivenom, and IC(a_n_) is the IC_50_ value of CTX A3 neutralized by a_n_ amount of antivenom.

### 4.9. Peptide Synthesis

Overlapping 15-mer synthetic peptides corresponding to the matured sequence of sNTX (accession: P60770) and CTX A3 (accession: P60301) were synthesized by the solid-phase method using a Prelude automated peptide synthesizer (Protein Technologies, Inc., Tucson, AZ, USA). In the process, α-amino groups were protected by the fluorenylmethoxycarbonyl group and the final de-blocking step was carried out with a mixture of TFA/triisopropylsilane/phenol/water/1,2-ethanedithiol. The crude peptide was precipitated with ether and further purified by reverse-phase HPLC. Purity was ensured to be higher than 90% by optical detector (UV 214 nm) performed on an Agilent 1100 Series LC/MSD high-performance ion-trap mass spectrometer.

### 4.10. Peptide ELISA

The reactivity of antibody to synthetic peptide was detected by ELISA following a procedure described previously [23]. In brief, a 96-well microtiter plate (Corning) was coated with 0.5 μg of individual synthetic peptide dissolved in 50 μL carbonate buffer (50 mM, pH 9.6) at 4 °C and incubated overnight. Plates were washed with PBS containing 0.05% (v/v) Tween 20 (PBST) and blocked with 5% skim milk in PBS at room temperature for 2 h. Antivenom (100 μg/mL in PBS/1% bovine serum albumin) was dispensed in each well and incubated at room temperature for 2 h. The plates were then washed four times with PBST and incubated with goat anti-horse IgG (1:10,000; Jackson ImmunoResearch Labs, West Grove, PA, USA) conjugated with horseradish peroxidase (HRP) for 1 h at room temperature. The assay was developed with the SureBlue Reserve™ TMB (3,3’,5,5’-tetramethylbenzidine) microwell peroxidase substrate (KPL, Gaithersburg, MD, USA) for 20 min, then stopped by adding 50 μL sulfuric acid (2 M H_2_SO_4_) solution. Absorbance at 450 nm (OD_450_) was measured using a Sunrise microplate reader. Two peptides, TFFLTQGALLNDK (TFF) and GILGFVFTLTVPSER (GIL), derived from influenza virus, were included as negative controls in the peptide ELISA assay. The assay was performed in triplicate for each peptide, and an unpaired Student’s *t*-test (two-tailed) was carried out to calculate the significance of peptide reactivity.

### 4.11. Determination of ELISA Antibody Titer against Immunoreactive Peptides

Serial-diluted antivenom (100 μL in PBS/1% BSA, pH 7.5) solutions were added to wells coated with either 0.5 μg of sNTX_21–35_ or CTX A3_43–57_ and incubated at room temperature for 2 h. The plates were then washed four times with PBST and incubated with either goat anti-horse IgG (1:10,000) or goat anti-mouse IgG (1:5000; GeneTex Inc., Irvine, CA, USA) conjugated with HRP for 1 h at room temperature. The assay was developed with TMB for 20 min, then stopped by adding 50 μL of sulfuric acid solution. Absorbance at 450 nm was measured by microplate reader, and ELISA endpoint titers were obtained from the titration curve by interpolation and defined as the reciprocal of serum dilution that maintained an OD_450_ value of 0.3. If OD_450_ value was <0.3 at the starting dilution or >0.3 at the final dilution, titers were obtained by extrapolation. All measurements were done in triplicate and the results are shown as mean ± standard deviation (SD).

### 4.12. sNTX_21–35_ Peptide Competitive-Binding Block Assay

To block peptide-specific antibodies, a constant amount of BAV was mixed with 10- and 30-fold molar amounts of sNTX_21–35_ peptide at 37 °C for 30 min. The neutralizing potency of peptide-blocked antivenom against the venom lethality was then assessed using an in-vivo animal assay as described in Section 4.6.3.

### 4.13. CTX A3_43–57_ Peptide-Competitive Binding Block Assay

A constant amount of SAV-Naja was mixed with 10- to 60-fold molar amounts of CTX A3_43–57_ peptide and incubated at 37 °C for 30 min. Then, 2.4 μg of CTX A3 was preincubated with 100 μg of peptide-blocked antivenom at 37 °C for 30 min before being treating with HL-60 cells. The antibody/peptide/CTX A3 mixture was added to a 96-microwell plate containing 1 × 10^5^ HL-60 cells and co-cultured for 4 h at 37 °C with 5% CO_2_. Subsequently, 20 μL of MTT solution (5 mg/mL) was added to each well and incubated for another 4 h. After removing the medium from the wells, 100 μL of dimethyl sulfoxide was added to dissolve formazan crystals, and the viability of cells was determined by detecting absorbance at 570 nm.

### 4.14. Statistical Analysis

One-way analysis of variance followed by Fisher’s least-significant difference test was carried out to compare the differences in cell viability. Statistical analysis of the neutralizing potencies of antivenom was performed by one-way analysis of variance followed by Bonferroni’s multiple comparisons test. An unpaired two-tailed *t*-test was carried out to compare the differences in antibody titers. Results with *p*-values < 0.05 were considered significant. All statistical analyses were performed using GraphPad Prism software.

## Figures and Tables

**Table 1:** Overview of the antivenomic data and the toxicities of venom components.

Protein	Percentage of Retained Protein ^b^ (%)			LD_50_ (μg/g)	IC_50_ (μg/mL)
	Antivenomic				
	BAV	SAV-Naja	NPAV		
sNTX	100.0	32.5	21.8	0.23 (0.17–0.31)	N.D. ^d^
CTXs ^a^	19.9	50.2	9.6	2.12 (1.82–2.49)	6.82 (5.99–7.76)
PLA_2_	17.6	74.2	100.0	>50 ^c^	N.D.

^a^ CTXs was a mixture of CTX A5, A1, A3 and A6 as indicated in Figure S1. ^b^ The percentage of retained protein (%) represented the extent of protein binding to antivenom-immobilized affinity column, which was calculated using the equation as described in experimental Section 4.3. ^c^ All the mice (*n* = 6, ICR, averaged weight 20 g) survived with the injection dosage of 50 μg purified PLA_2_ molecule. ^d^ N.D.: not detected.

**Table 2:** List of the neutralization potency of antivenoms against either the lethal effect of *N. atra* venom or the cytotoxic activity of CTX A3.

Antivenom	5 × LD_50_ *N. atra* Venom			CTX A3
	ED_50_ (mg) ^a^	ER_50_ (mg/g) ^b^	P (mg/g) ^c^	P_c_ (mg/g) ^d^
BAV	0.66	101.82 (86.97–119.17)	81.5	3.87 *
SAV-Naja	3.86	17.41 (14.87–20.38)	13.9	9.40 ^§^
NPAV	6.93	9.70 (8.28–11.35)	7.8	NE
DAV	NE ^e^	NE	NE	NE

^a^ ED_50_: median effective dose, the amount of antivenom protecting the 50% of mice that survived under the challenge dosage of 5 × LD_50_ *N. atra* venom. ^b^ ED_50_: effective dose ratio, the amount of venom neutralized per gram of antivenom at which 50% of challenged mice survived. ^c^ P: potency, defined as the amount of venom neutralized per gram of antivenom. ^d^ P_c_: potency against cytotoxicity, defined as the amount of CTX A3 neutralized per gram of antivenom. ^e^ NE displays ineffective. * Significantly different (*p* < 0.05) from the potency of DAV antivenom. ^§^ Significantly different (*p* < 0.0001) from the potency of BAV antivenom.

## Supplementary Materials

The following are available online at www.mdpi.com/2072-6651/10/1/10/s1, Figure S1: Evaluation of antivenom efficacy in capturing *N. atra* venom components. (**A**) High-performance liquid chromatography (HPLC) chromatogram of 500 μg crude venom of *N. atra*. The components within the chromatographic peaks (nos. 1–10) were identified using LC–MS/MS (Table S1). The elution (Elu) and flow through (FT) fractions of (**B**,**C**) bivalent antivenom (BAV)-, (**D**,**E**) snake antivenom-Naja (SAV-Naja)-, and (**F**,**G**) neuro polyvalent snake antivenom (NPAV)-immobilized affinity columns were collected and analyzed by reverse-phase HPLC to determine the retained percentage of venom components, Figure S2: Analyzing the cytotoxicity of CTX analogs of *N. atra* venom using the cell-based assay, Figure S3: Reverse-phase HPLC chromatograms of 300 μg of (**A**) *N. atra* and (**B**) *N. kaouthia* venom used in this study, Table S1: Identification of *N. atra* venom components within the HPLC chromatographic fractions using LC-MS/MS, Table S2: List of synthetic peptides used for the immunoreactive peptide mapping study.
