# Supplementary material for: Identification of Immunoreactive Peptides of Toxins to Simultaneously Assess the Neutralization Potency of Antivenoms against Neurotoxicity and Cytotoxicity of Naja atra Venom
Source: Toxins (Basel). 2017 Dec 25;10(1):10. doi: 10.3390/toxins10010010 (PMC5793097; doi:10.3390/toxins10010010)
Supplement: Supplementary file 1 [file toxins-10-00010-s001.pdf]

Supplementary Materials: Identification of Immunoreactive Peptides of Toxins to Simultaneously Assess the Neutralization Potency of Antivenoms against Neurotoxicity and Cytotoxicity of *Naja atra* Venom

Bing-Sin Liu, Wen-Guey Wu, Min-Han Lin, Chi-Han Li, Bo-Rong Jiang, Suh-Chin Wu, Chih- Hsiang Leng and Wang-Chou Sung

Table S1. Identification of *N. atra* venom components within the HPLC chromatographic fractions using LC-MS/MS.

[a]Peak	Protein identity	[b]AC no.	Significant peptide	Ion, m/z	MS score	Toxin family	[c]Toxicity score
1	Cobrotoxin<br>-	P60770	K.NGIEINCCTTDR.C<br>R.GCGCPSVK.N	726.7767 (2+)<br>432.6666 (2+)	88	3FTX	60.3
2	Cobrotoxin-b	P80958	K.VKPGVNLNCCTTDR.C<br>K.TCSGETNCYK.K<br>K.TCSGETNCYKK.W	545.2432 (3+)<br>610.2364 (2+)<br>674.2805 (2+)	170	3FTX	5.0
3	Phospholipase A_2	P00598	R.SWWDFADYGCYCGR.G<br>R.LAAICFAGAPYNNNNYNID<br>LK.AR.CCQVHDNCYNEAEK.I<br>K.TYSYECSQGTLTCK.G<br>K.GGNNACAAAVCDCDR.L<br>K.NMIQCTVPSR.S<br>R.GGSGTPVDDLDR.C<br>K.ISGCWPYFK.T	921.8533 (2+)<br>1178.5278 (2+)<br><br>609.5383 (2+)<br>849.3058 (2+)<br>805.7562 (2+)<br><br>603.2667 (2+)<br>594.7442 (2+)<br>579.2611(2+)	859	PLA_2	N.D.
4	Cardiotoxin A5	P62375	K.CHNTQLPFIYK.T<br>K.YVCCSTDKCN.-<br>R.GCADNCPK.N<br>K.FPLKFPVK.R<br>K.YVCCSTDK.C	710.8403 (2+)<br>653.7474 (2+)<br>461.1946 (2+)<br>488.3006 (2+)<br>516.6811 (2+)	163	3FTX	
5	Cardiotoxin A1	P60304	K.MFMMSDLTIPVK.R<br>K.LIPIASK.T<br>R.GCIDVCPK.N<br>K.YVCCNTDR.C<br>K.RGCIDVCPK.N<br>K.MFMMSDLTIPVKR.G	706.8523 (2+)<br>371.2426 (2+)<br>474.6818 (2+)<br>544.2131(2+)<br>552.7282 (2+)<br>523.5909 (3+)	308	3FTX	25.95
6	Cardiotoxin A3	P60301	K.LVPLFYK.T<br>K.MFMVATPK.V<br>K.MFMVATPK.V	440.2615 (2+)<br>462.7289 (2+)<br>478.7285 (2+)	274	3FTX	
7	Cardiotoxin A6	P80245	K.MFMVAAPK.V<br>K.CNQLIPPFYK.A	447.7211 (2+)<br>640.3278 (2+)	284	3FTX	
8	Cysteine-rich secretory protein	Q7T1K6	R.WANTCSLNHSPDNLR.V<br>R.AGCAVSYCPSSAWSYFYVCQ<br>YCPSGNFQGK.T<br>R.VSPTASNMLK.M<br>K.LTNCDSLLK.Q<br>K.EIVDLHNSLR.R<br>K.SNCPASCFCR.N<br>K.QSSCQDDWIK.S	595.5896 (3+)<br>1167.8121 (3+)<br><br>524.2760 (2+)<br>532.2622 (2+)<br>598.3191 (2+)<br>629.7216 (2+)<br>633.7682 (2+)	223	CRISP	N.D.
9	Zinc metalloproteinase-disintegrin-like kaouthiagin-like	D3TTC1	R.VAKDDCDLPELCTGQSAECP<br>TDSLQR.N<br>R.NDNAQLLTGIDFNGNTVGR.<br>A<br>K.FEVKPAASVTLK.S<br>R.TAPAFQFSSCSIR.E<br>K.DKFEVKPAASVTLK.S	989.0843 (3+)<br><br>1009.9933 (2+)<br><br>430.5788 (3+)<br>736.3360 (2+)<br>511.6191 (3+)	192	SVMP	N.D.

			K.ASCICIPGPCIMLK.K	810.3913 (2+)			
10	Zinc metalloproteinase-disintegrin-like atragin	D3TTC2	R.AAKDDCDLPELCTGQSAECPTDVFQR.N	995.0845 (3+)	281	SVMP	N.D.
			K.TSAAVVQDYSSR.T	642.3005 (2+)			
			R.KIPCAAK.D	394.2237 (2+)			
			R.GFCTCGFNK.C	545.7274 (2+)			
			R.DSCFTLNQR.T	570.7505 (2+)			
			R.ATLNLFGEWR.E	603.8043 (2+)			
			R.TKPAYQFSSCSVR.E	765.8667 (2+)			
			K.LQHEAQCDSEECCEK.C	641.5699 (3+)			
			K.DDCDLPELCTGQSAECPTDVFQR.N	905.0342 (3+)			

[a] The peak refers to the peak number highlighted in Figure S1. [b] AC is the abbreviation of accession number in Uniprot database. [c] Toxicity score was calculated according to Laustsen, A et al. [25] by the ratio of protein abundance (%) estimated from the reverse phase HPLC chromatography to its medium lethal dose (LD$_{50}$). The toxicity score of the crude venom, which abundance was defined as 100%, was 149.3. N.D. represented LD$_{50}$ was undetectable at a cut-off value of 50 μg per mouse. Abbreviations: SVMP indicates snake venom metalloproteinase. CRISP is cysteine-rich secretory protein. 3FTX means three finger toxins.

Table S2. List of synthetic peptides used for the immunoreactive peptide mapping study.

Index	Peptide sequence
CTXA3$_{1-15}$	LKCNKLVPLFYKTCP
CTXA3$_{5-19}$	KLVPLFYKTCPAGKN
CTXA3$_{11-25}$	YKTCPAGKNLCYKMF
CTXA3$_{15-29}$	PAGKNLCYKMFMVAT
CTXA3$_{21-35}$	CYKMFMVATPKVPVK
CTXA3$_{26-40}$	MVATPKVPVKRGCID
CTXA3$_{31-45}$	KVPVKRGCIDVCPKS
CTXA3$_{36-50}$	RGCIDVCPKSSLLVK
CTXA3$_{43-57}$	PKSSLLVKYVCCNTD
CTXA3$_{46-60}$	SLLVKYVCCNTDRCN
sNTX$_{1-15}$	LECHNQQSSQTPTTT
sNTX$_{4-18}$	HNQQSSQTPTTTGCS
sNTX$_{11-25}$	TPTTTGCSGGETNCY
sNTX$_{16-30}$	GCSGGETNCYKKRWR
sNTX$_{21-35}$	ETNCYKKRWRDHRGY
sNTX$_{26-40}$	KKRWRDHRGYRTERG
sNTX$_{31-45}$	DHRGYRTERGCGCPS
sNTX$_{36-50}$	RTERGCGCPSVKNGI
sNTX$_{39-53}$	RGCGCPSVKNGIEIN
sNTX$_{45-59}$	SVKNGIEINCCTTDR
sNTX$_{48-62}$	NGIEINCCTTDRCNN
[a] TFF	TFFLTQGALLNDK
[b] GIL	GILGFVFTLTVPSER

[a] TFF is the abbreviation of TFFLTQGALLNDK which is a partial sequence of neuraminidase of H1N1 influenza virus. [b] GIL is the abbreviation of GILGFVFTLTVPSER which is a partial sequence of M1 protein of H5N1 influenza virus.

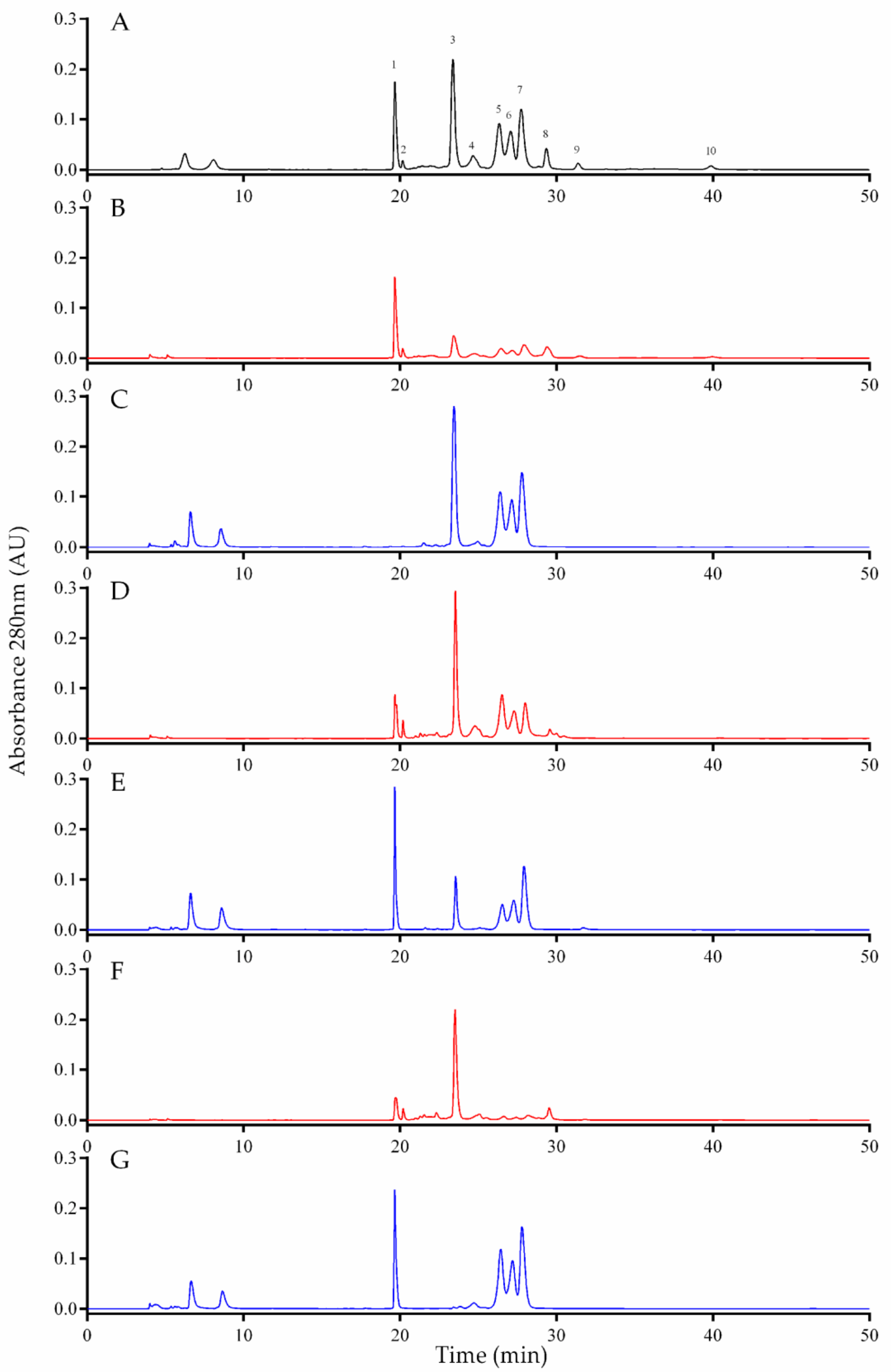

Figure S1. Evaluation of antivenom efficacy in capturing *N. atra* venom components. (**A**) HPLC chromatogram of 500 µg of *N. atra* crude venom. The component within the chromatographic peaks (no.1-10) were identified using LC MS/MS (Table S1). The elution (Elu) and flow through (FT) fractions of (**B**,**C**) BAV-, (**D**,**E**) SAV-Naja-, and (**F**,**G**) NPAV-immobilized affinity columns were collected and analyzed by reverse phase HPLC to determine the retained percentage of venom components.

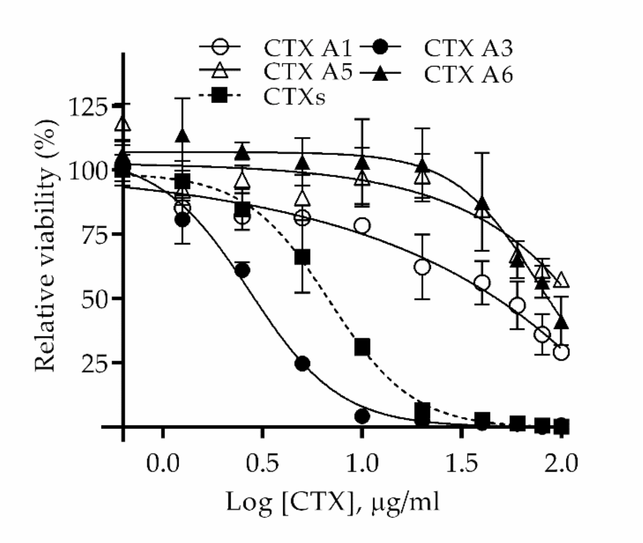

Figure S2. Analyzing the cytotoxicity of CTX analogs of *N. atra* venom using the cell-based assay.

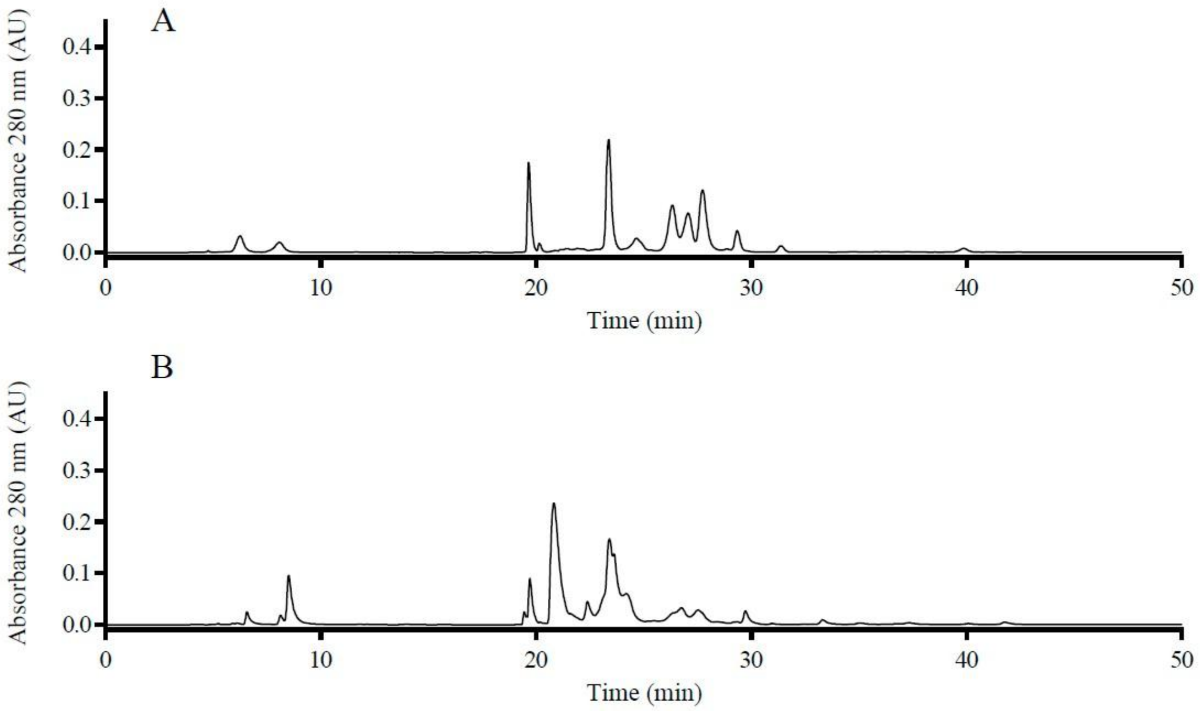

Figure S3. Reverse phase HPLC chromatograms of 300 μg of (**A**) *N. atra* and (**B**) *N. kaouthia* venom used in this study.
